# Does mRNA SARS-CoV-2 vaccine influence patients' performance during IVF-ET cycle?

**DOI:** 10.1186/s12958-021-00757-6

**Published:** 2021-05-13

**Authors:** Raoul Orvieto, Meirav Noach-Hirsh, Aliza Segev-Zahav, Jigal Haas, Ravit Nahum, Adva Aizer

**Affiliations:** 1 Department of Obstetrics and Gynecology, Chaim Sheba Medical Center (Tel Hashomer), Ramat Gan, Israel; 2 Sackler Faculty of Medicine, Tel Aviv University, Tel Aviv, Israel; 3 The Tarnesby-Tarnowski Chair for Family Planning and Fertility Regulation, Sackler Faculty of Medicine, Tel-Aviv University, Tel Aviv, Israel

**Keywords:** COVID-19, vaccination, Ovarian stimulation, embryo quality, IVF

## Abstract

**Objective:**

No information exists in the literature regarding the effect of mRNA SARS-CoV-2 vaccine on subsequent IVF cycle attempt. We therefore aim to assess the influence of mRNA SARS-CoV-2 vaccine on IVF treatments.

**Design:**

An observational study.

**Setting:**

A tertiary, university-affiliated medical center.

**Patients and Methods:**

All couples undergoing consecutive ovarian stimulation cycles for IVF before and after receiving mRNA SARS-CoV-2 vaccine, and reached the ovum pick-up (OPU) stage. The stimulation characteristics and embryological variables of couples undergoing IVF treatments after receiving mRNA SARS-CoV-2 vaccine were assessed and compared to their IVF cycles prior to vaccination.

**Main outcome measures:**

Stimulation characteristics and embryological variables.

**Results:**

Thirty-six couples resumed IVF treatment 7–85 days after receiving mRNA SARS-CoV-2 vaccine. No in-between cycles differences were observed in ovarian stimulation and embryological variables before and after receiving mRNA SARS-CoV-2 vaccination.

**Conclusions:**

mRNA SARS-CoV-2 vaccine did not affect patients’ performance or ovarian reserve in their immediate subsequent IVF cycle. Future larger studies with longer follow-up will be needed to validate our observations.

## Introduction

Coronavirus disease 19 (COVID-19) emerged in Wuhan, Hubei province, China [1] in December 2019, and rapidly spread worldwide, affecting millions of people, with more fatalities compared with the SARS and MERS coronavirus epidemics combined.

When considering the relationship between COVID-19 infection and infertility or infertility treatments, the ASRM Coronavirus/COVID-19 Task Force [2] emphasized that the existing evidence suggests that “the virus likely does not infect gametes [3, 4] or embryos”, although no information exists in the literature regarding the influence of COVID-19 infection on laboratory/ embryological variables nor ovarian stimulation (OS) during the subsequent in-vitro fertilization (IVF) cycle attempt- which is considered the " most reliable sign of decrease ovarian reserve” [5].

Recently, we assessed the influence of COVID-19 infection on the stimulation characteristics and embryological variables of patients’ IVF treatments, before and after recovering from COVID-19 infection [6]. COVID-19 infection did not affect patients’ performance or ovarian reserve in their immediate subsequent IVF cycle, except for a reduced proportion of top quality embryos (TQEs).

The newly available mRNA SARS-CoV-2 vaccine by Pfizer has been shown to be 95 % effective in preventing SARS-CoV-2 infection a week following the second dose, with a favorable safety profile in a 2-month median follow up time [7]. It was shown to elicit high SARS-COV2 neutralizing antibody titers alongside high antigen specific CD8 + and Th1 type CD4 + T cell response. Prompted by the aforementioned observations, unfounded claims in the popular media linked a possible correlation between the SARS-CoV-2 vaccine and potential infertility. Currently, there is no information in the medical literature to confirm or dispute these unfounded claims. The aim of this observational study was to investigate the effect of BNT162b2 SARS-CoV-2 vaccination on OS characteristics and the embryological variables during the IVF treatment post COVID-19 infection, in order to aid both fertility specialists counselling and their patients in their decision-making process.

### Patients and methods

The study population consisted of all couples undergoing consecutive OS for IVF, before and following the second dose of the vaccination, and reached the ovum pick-up (OPU) stage. The study was approved by the institutional research ethics board of Sheba Medical Centre.

Data on patient age and infertility-treatment-related variables were collected from the files. Embryological/laboratory variables of the IVF cycles were assessed and compared between the patients’ IVF cycle before and following the second dose of the vaccination. Embryos classification was based on the individual embryo scoring parameters according to pre-established definitions [8]. A TQE was defined as seven or more blastomeres on day 3, equally-sized blastomeres and ≤ 10 % fragmentation.

Following a positive pregnancy test, ongoing pregnancies were confirmed by presence of gestational sac with fetal heart rate on ultrasound at 6–8-week gestation.

Statistical analysis was performed with paired-Student’s t-test and Chi square, as appropriate. Results are presented as means ± standard deviations; p < 0.05 was considered significant.

## Results

Of all couples who underwent IVF cycle treatments in our centre before the COVID-19 pandemic, in 36, both partners received the two doses of the vaccination. None of the couples suffered from co-morbidities (e.g. diabetes, obesity, HTN, asthma, cardiac disease). The interval between the time of the second vaccine to the date of the subsequent IVF treatment cycle was 7–85 days.

Patients clinical characteristics and the details of their IVF cycle attempts, before and after the mRNA SARS-CoV-2 vaccine, are shown in Tables 1 and 2. There were no differences between the cycles in the length of OS, total dose of gonadotropin used, nor the peak estradiol and progesterone levels (Table 2).

**Table 1:** Patients’ baseline clinical characteristics

	Female	Male
Number of patients	36	36
Mean interval between OPU cycles / Sperm test (months)	5.2 ± 6.1	4.2 ± 4.9
(Range)	(1–24)	(0–23)
Mean interval between Second Vaccination to OPU cycle / Sperm test (days)	32.6 ± 17.5	33.3 ± 14.9
(Range)	(7–85)	(7–85)
Age, years (mean ± SD)	37.3 ± 4.6	40.1 ± 4.8
Gravida (mean ± SD)	2 ± 1.5	---
Para (mean ± SD)	0.9 ± 0.9	---
Smoking (%)	3/36 (8.3)	---
BMI, Kg/m^2^ (mean ± SD)	26.3 ± 5.4	---
Mean basal FSH IU/L (mean ± SD)	8.2 ± 3.8	---
Mean basal LH IU/L (mean ± SD)	5.1 ± 2.5	---
**Type of infertility**		
Male (%)	6/36 (16.7)	
Tubal (%)	1/36 (2.8)	
Endometriosis (%)	4/36 (11.1)	
Unexplained infertility (%)	4/36(11.1)	
Ovulatory disorder (%)	1/36 (2.8)	
Uterine factor (%)	1/36 (2.8)	
Others (%)	26/36 (72.2)

**Table 2:** Patients’ OS variables and IVF cycle laboratory characteristics Pre/Post Covid-19 Vaccination

	Pre Vaccination	Post Vaccination	*P*-value
Antagonist Protocol (%)	34/36 (94.4)	35/36 (97.2)	NS
Duration of stimulation (days)	10.7 ± 2.6	10.8 ± 2.2	NS
Total FSH dose used, IU (mean ± SD)	3802 ± 1956	3906 ± 1565	NS
Mean peak estradiol levels pmol/L (mean ± SD)	6041 ± 4052	7708 ± 7640	NS
Mean peak progesterone levels nmol/L (mean ± SD)	2.3 ± 1.8	2.2 ± 1.2	NS
Mean # of Oocytes per OPU (mean ± SD)	9.7 ± 6.7	10.1 ± 8	NS
Mean # of MII per OPU (mean ± SD)	7.94 ± 5.7	8.0 ± 6.5	NS
Mean # of MII / # of oocytes retrieved (mean ± SD)	0.83 ± 0.1	0.78 ± 0.2	NS
Mean # of 2PN per OPU (mean ± SD)	6.44 ± 5.0	6.5 ± 5.8	NS
Mean # of 2PN/ # of oocytes retrieved (mean ± SD)	0.69 ± 0.2	0.63 ± 0.2	NS
Mean # of TQE per OPU (mean ± SD)	2.8 ± 2.7	2.8 ± 3.3	NS
Mean # of TQE/ # of 2PN(mean ± SD)	0.40 ± 0.3	0.40 ± 0.2	NS
Semen volume (mL) (mean ± SD)	3.0 ± 1.4	3.2 ± 1.7	NS
Sperm concentration (Millions)(mean ± SD)	72.4 ± 61.5	80.2 ± 55.7	NS
Sperm % motility (mean ± SD)	56 ± 22	54 ± 20	NS
Pre-wash total motile sperm count, millions (mean ± SD)	134 ± 169	146 ± 159	NS

Furthermore, no differences were observed in the number of oocytes and mature oocytes retrieved, fertilization rate, TQE and the ratio of TQEs per number of 2PN, or semen analyses (Table 2).

No patients conceived in the IVF treatment cycle before receiving the vaccine, while 3 pregnancies were recorded in the 10 patients who underwent embryo transfer (30 % per transfer) in the cycle following mRNA SARS-CoV-2 vaccination.

## Discussion

In the present study we observed no influence of mRNA SARS-CoV-2 vaccine on patients’ performance during their immediate subsequent IVF cycle, reflecting no detrimental effects of the vaccine on patients’ ovarian reserve, nor the developing gametes/embryos, with an acceptable pregnancy rate (30 % per transfer).

To date, damage to the female reproductive system in COVID-19 patients has not been reported. There is indirect evidence that COVID-19 might affect female fertility by attacking ovarian tissue and granulosa cells, and decreasing ovarian function and oocyte quality. Moreover, COVID-19 might damage endometrial epithelial cells and affect early embryo implantation [9–12]. A recent study by our group could not demonstrate any effect of COVID-19 infection on the OS characteristics and embryological variables of patients’ IVF treatments, except for a reduced proportion of top quality embryos.

Since folliculogenesis and spermatogenesis are complex and dynamic processes involving multiple endocrine cells and numerous signals that have been estimated to span > 3 months [13, 14]. The COVID-19 infection, by its known ability to activate the release large amounts of pro-inflammatory cytokines and precipitate and sustain an aberrant systemic inflammation [15], might also interfere with these processes, resulting in abnormal gametes (oocytes and sperms), with the consequent production of low quality embryos.

Following mRNA SARS-CoV-2 vaccine, we could not observe any detrimental effect on OS characteristics, embryological variables nor the proportion of top quality embryos. These might be explained by the lesser degree of systemic inflammation induced by the vaccine, with modest effect on folliculogenesis and spermatogenesis.

In the present study, we could not demonstrate any detrimental effect of mRNA SARS-CoV-2 vaccine on ovarian reserve/ oocytes pool, as reflected by the similar response to OS- which is considered the " most reliable sign of decrease ovarian reserve [5]. Moreover, since the IVF treatment attempts were conducted 7–85 days post vaccination, when the retrieved gametes during these cycles were exposed to the mRNA SARS-CoV-2 vaccine induced systemic inflammation during their development, in contrast with active infection [6], any potential inflammatory environment following the vaccine did not interfere with the intricate complex processes of folliculogenesis and spermatogenesis.

Regarding the effect of COVID-19 on the male reproductive system, this issue is even more controversial. While 5 studies failed to detect the presence of COVID-19 viral RNA in the semen samples of patients with active or resolving infection [16–20], one study identified COVID-19 RNA in 15.38% of the semen samples [21] and another study [20] demonstrated that patients with moderate infection had significantly reduced sperm quantity and quality, compared to patients with mild infection or normal controls. In the present study, mRNA SARS-CoV-2 vaccine showed no detrimental effect on patients' total motile count.

The limitations of our study is the small sample size and the short period of follow-up. A major strength of our study is that we compared two consecutive IVF cycle attempts (before and following vaccination) in the same cohort of patients. The fact that all women that participated in our study had two consecutive treatment cycles, helps to eliminate multiple bias factors and to attribute the study results to the pre and post vaccination effect.

In conclusions, mRNA SARS-CoV-2 vaccine did not affect patients’ performance or ovarian reserve in their immediate subsequent IVF cycle. Future larger studies with longer follow-up will be needed to validate our observations.

## Data Availability

N/A.

## Abbreviations

COVID-19: Coronavirus disease 19.
IVF: In-vitro fertilization.
OPU: Ovum pick-up.
OS: Ovarian stimulation.
TQE: Top quality embryos.
